# *In silico* molecular cytogenetics: a bioinformatic approach to prioritization of candidate genes and copy number variations for basic and clinical genome research

**DOI:** 10.1186/s13039-014-0098-z

**Published:** 2014-12-09

**Authors:** Ivan Y Iourov, Svetlana G Vorsanova, Yuri B Yurov

**Affiliations:** Mental Health Research Center, Russian Academy of Medical Sciences, 117152 Moscow, Russia; Russian National Research Medical University named after N.I. Pirogov, Separated Structural Unit “Clinical Research Institute of Pediatrics”, Ministry of Health of Russian Federation, 125412 Moscow, Russia; Department of Medical Genetics, Russian Medical Academy of Postgraduate Education, Moscow, 123995 Russia

**Keywords:** Bioinformatics, Candidate genes, Chromosome imbalances, Copy number variation, Gene expression, Molecular cytogenetics, Somatic mosacism

## Abstract

**Background:**

The availability of multiple *in silico* tools for prioritizing genetic variants widens the possibilities for converting genomic data into biological knowledge. However, in molecular cytogenetics, bioinformatic analyses are generally limited to result visualization or database mining for finding similar cytogenetic data. Obviously, the potential of bioinformatics might go beyond these applications. On the other hand, the requirements for performing successful *in silico* analyses (i.e. deep knowledge of computer science, statistics etc.) can hinder the implementation of bioinformatics in clinical and basic molecular cytogenetic research. Here, we propose a bioinformatic approach to prioritization of genomic variations that is able to solve these problems.

**Results:**

Selecting gene expression as an initial criterion, we have proposed a bioinformatic approach combining filtering and ranking prioritization strategies, which includes analyzing metabolome and interactome data on proteins encoded by candidate genes. To finalize the prioritization of genetic variants, genomic, epigenomic, interactomic and metabolomic data fusion has been made. Structural abnormalities and aneuploidy revealed by array CGH and FISH have been evaluated to test the approach through determining genotype-phenotype correlations, which have been found similar to those of previous studies. Additionally, we have been able to prioritize copy number variations (CNV) (i.e. differentiate between benign CNV and CNV with phenotypic outcome). Finally, the approach has been applied to prioritize genetic variants in cases of somatic mosaicism (including tissue-specific mosaicism).

**Conclusions:**

In order to provide for an *in silico* evaluation of molecular cytogenetic data, we have proposed a bioinformatic approach to prioritization of candidate genes and CNV. While having the disadvantage of possible unavailability of gene expression data or lack of expression variability between genes of interest, the approach provides several advantages. These are (i) the versatility due to independence from specific databases/tools or software, (ii) relative algorithm simplicity (possibility to avoid sophisticated computational/statistical methodology) and (iii) applicability to molecular cytogenetic data because of the chromosome-centric nature. In conclusion, the approach is able to become useful for increasing the yield of molecular cytogenetic techniques.

## Background

To produce biological knowledge on the basis of high-throughput analyses of genome, *in silico* methods are required. Technical resource limitations in acquiring and validating data on mechanisms and consequences of genetic variants suggest the robust selection to underlie the associations with phenotypic traits. Consequently, candidate gene prioritization seems to represent a valuable approach to validate genomic associations *in silico* and, more importantly, to exacerbate the significance of molecular findings [1,2]. Actually, functional characteristics of genes seem to be the most useful parameters for establishing genetic associations [3-6]. However, there is a strong evidence from molecular cytogenetic studies that copy numbers of genes involved in a variety of critical biological processes can be variable without apparent phenotypic effect [7,8]. Therefore, one can propose bioinformatic classification of genetic variants to be important for distinguishing between benign and pathogenic mutations.

Recently, several bioinformatic assays applicable to molecular cytogenetics have been described and have served as a basis to develop more sophisticated techniques to detect chromosomal rearrangements and to generalize genomic data [9-13]. Notwithstanding, bioinformatic methods are rarely used in molecular cytogenetic studies. Our own efforts in this regard have been made to define the consequences of genomic variations according to *in silico* surveying gene expression [14-16]. Despite this relative success, requirements of additional selection criteria and more detailed *in silico* analysis of genome (epigenome and proteome) data have been acknowledged. Here, incorporating several new features (selection criteria) and integration/fusion of data from multiple databases/resources, we propose a bioinformatic approach to prioritization of candidate genes and copy number variations (CNV). We further speculate that this approach can be useful for basic and applied molecular cytogenetic genome research.

## Results and discussion

### Gene expression as a criterion for the prioritization

The variability of expression profiles between genes located in a genomic locus in a given tissue is relatively stable. Moreover, a number of epigenetic databases (i.e. BioGPS [17]) provide rather visualization of such variability than gene-specific expression data. Using the same idea as proposed earlier for tissue-specific genome pathology (i.e. brain diseases should primarily result from genomic alterations affecting brain tissue) [18], we have hypothesized that a gene mutation (CNV/chromosome rearrangement) is likely to be associated with specific trait if the gene is expressed more abundantly in the affected tissue. Thus, our model suggests that a genetic variant is more likely to have a phenotypic outcome due to dysfunctions in specific tissues or cell lineages. The latter appears to be achieved through unequal distribution of gene expression patterns in different tissues. Hence, it becomes possible to attribute genes involved in a chromosome rearrangement or CNV to specific cellular processes or tissue pathology.

Gene expression has long been recognized as a valuable criterion of classifying cellular or pathological states and prioritizing genetic variants [19,20]. Furthermore, alterations to gene expression are associated with pathological conditions and are able to indicate changes in molecular pathways [21]. Therefore, gene expression may be appropriate as the second step in filtering strategy, following the first (empirical) filter of detecting genomic variations (molecular cytogenetic analysis of chromosome abnormalities or CNV) highlighting genes, which are to be analyzed bioinformatically. Nevertheless, to increase the efficiency of gene prioritization additional filters and ranking strategies are needed.

### From genes to pathways and back again

**Figure 1:**
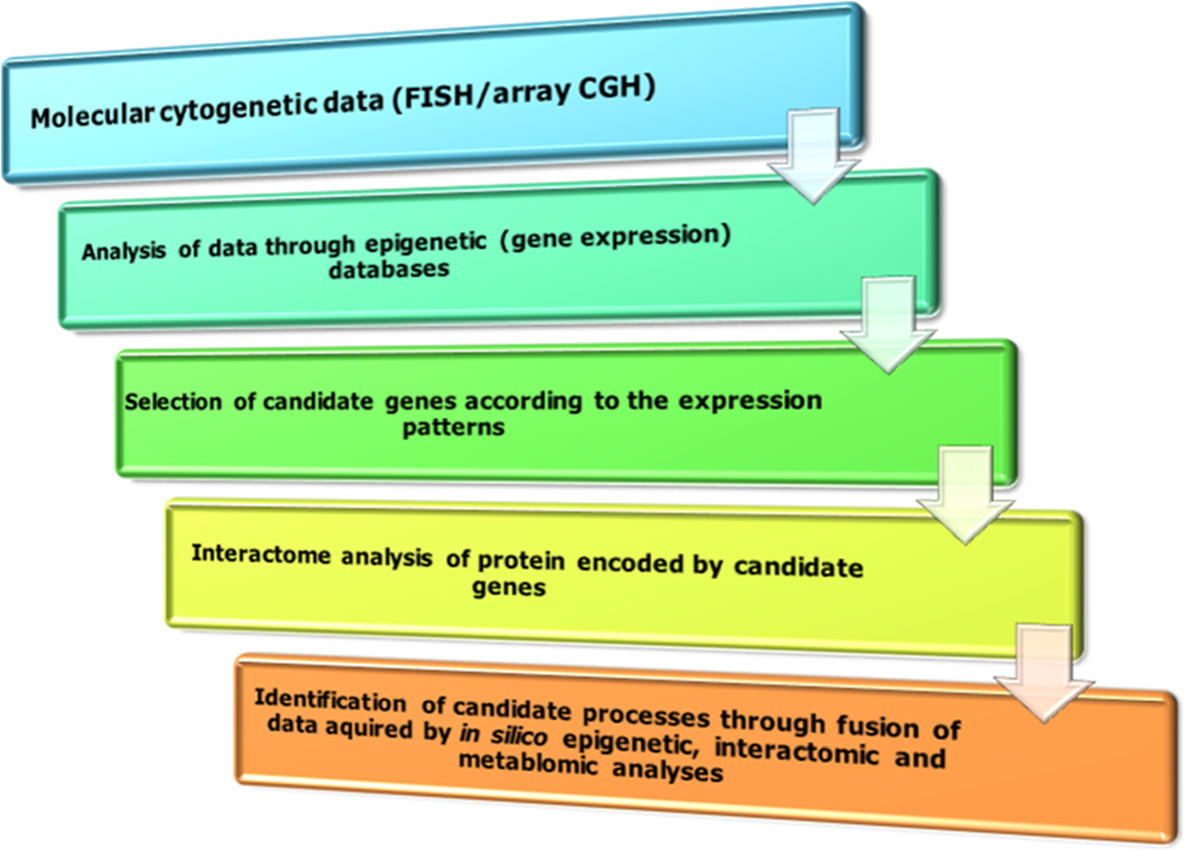
**Outline of the basic procedure: molecular cytogenetic data (i.e. genes involved in a chromosome imbalance) is analyzed using epigenetic (gene expression) databases.** According to epigenetic *in silico* analysis candidate genes are initially prioritized. Nextly, interactome analysis of proteins encoded by candidate genes is done. All these data is then fused for identification of disease candidate processes.

Filtering strategy based on analysis of genomic/epigenomic databases has been combined with a ranking strategy and other properties of selected genes (proteins), acquired from complementary data sources, have been considered to define the most promising candidates. The definitions of filtering and ranking strategies for data fusion have been previously described in Moreau and Tranchevent, 2012 [1]. Following gene selection according to their expression profiles, additional data was acquired from proteomic databases (i.e. consequences of gene mutations at protein level, interactions between proteins (interactome networks), pathways (“reactome”) and metabolic processes or metabolome). Moreover, genetic variants were addressed in Database of Genomic Variants (http://dgvbeta.tcag.ca). Using these data to associate genetic variability with phenotypic traits, it has become possible to identify candidate processes for a disease in addition to candidate genes. An outline of the procedure is given in Figure 1.

Specific interactomes and metabolomes can be used for prioritization of genes outside of chromosome imbalance or CNV. The scheme of such prioritization would look as follows: (i) genes involved in a genomic rearrangement (or mutated genes) are selected according to the gene expression profiles; (ii) data acquired from genomic/proteomic/metabolomic are used to construct the network or pathway specific for a clinical condition or phenotypic trait; (iii) other elements (genes) of this network/pathway are evaluated by the approach. Therefore, the applications of the approach are not limited to those genes involved in a chromosome imbalance or CNV.

Integrating multiple data sources is likely to be the most appropriate way to prioritizing genes using *in silico* techniques [22]. This can be done through the evaluation of ontology-based gene similarities [2], fusion of data from different resources [3], or analyzing of protein interaction networks (interactomes) [4,5]. In any case, results of bioinformatic analyses can be confirmed by a “clinical verification” or in other words, genotype-phenotype correlations. However, such verification has a disadvantage inasmuch as phenotypic outcomes can be intricate or can manifest later in life. Genetic variants causing susceptibility to complex diseases represent another problem regardless of the widespread ignorance in terms of CNV pathogenic value. Here, one has to consider the fact that this genetic problem has not as yet been solved in a satisfactory manner.

### Gene prioritization in chromosomal imbalances

**Figure 2:**
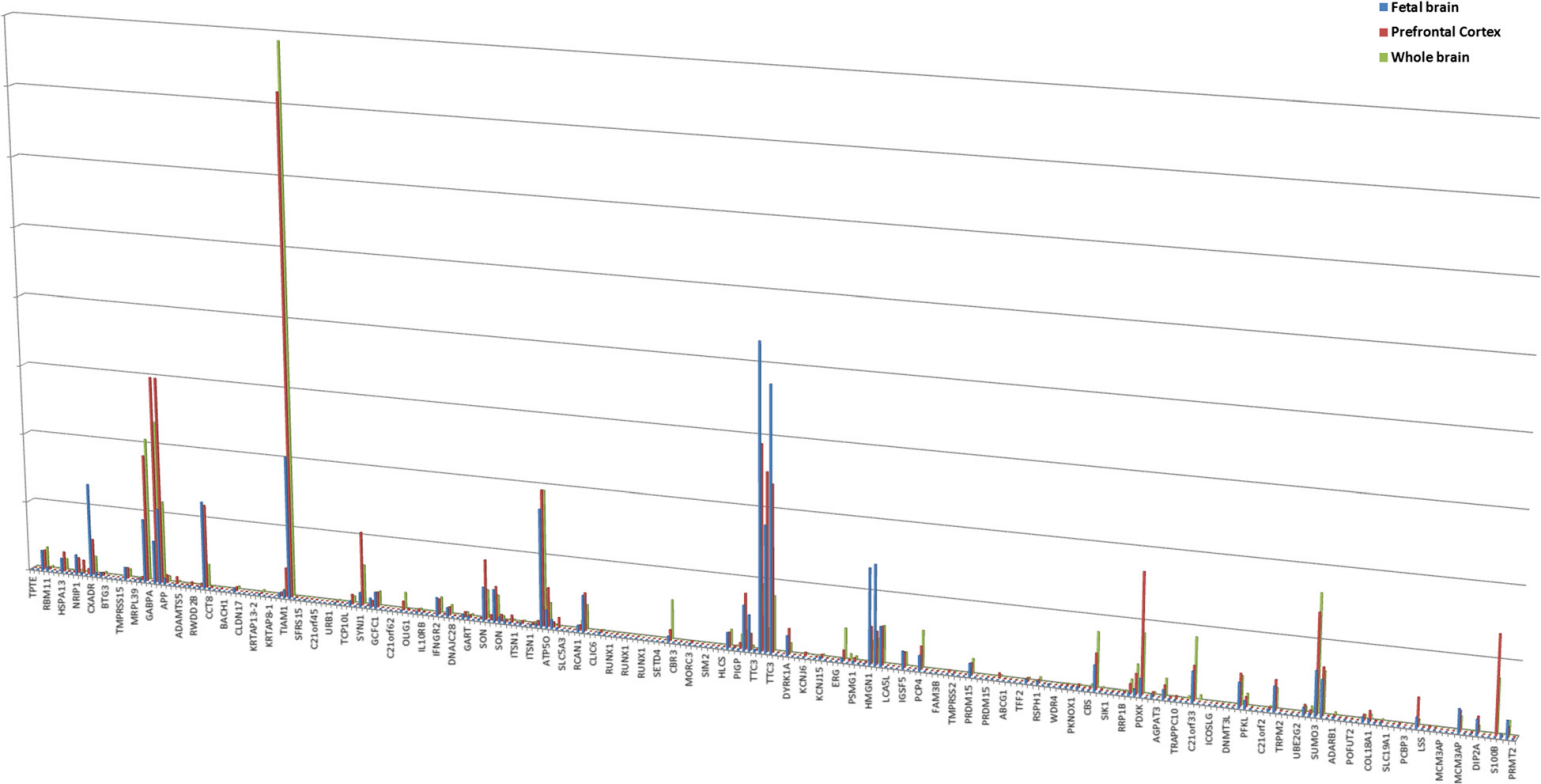
**Alignment of gene expression profiles in the fetal/whole brain and prefrontal cortex to chromosome 21 long arm.** Each expression profile (ordinates) was placed on the graph according to gene localization (abscissa) acquired from NCBI Map Viewer. Gene expression profiles were acquired from www.biogps.org [17].

Structural chromosomal imbalances and aneuploidy have been used as a model for gene prioritization because of recognizable phenotypes (ease of correlation between genotypes and phenotypes) and positive data on molecular definition of chromosomal syndromes. Taking the most prominent example of chromosomal imbalances referred to as trisomy of chromosome 21 or Down’s syndrome, we have tested the approach by analysis of candidate genes for brain malfunction in this devastating disease. Firstly, the alignment of gene expression profiles within fetal and adult brain tissues was made (Figure 2). The selected genes (outliers) (*CXADR, GABPA, APP, TIAM1, SYNJ1, SON, ATP5O, TTC3, HMGN1, PDXK, SUMO3, S100B*) were further evaluated using the ranking strategy. Comparing these data with those on brain dysfunction pathways in Down’s syndrome [23], we have found that disease networks matched the networks of the present study. Trisomy 21 provides an example how karyotype alterations achieve a broad impact on (cellular) phenotype, affecting simultaneously many genes and changing the expression of genes outside of chromosome 21 [18,23]. Accordingly, this is achieved through alterations to several pathways, among which are chromatin remodeling and gene expression regulation. Apparently, the way these pathways are altered is likely to be properly evaluated by *in silico* molecular cytogenetic approaches. Although these results were generally expected, one can agree that this testing shows the applicability of the bioinformatic approach. We also have performed bioinformatic analysis of two cases of terminal 7q loss detected by array comparative genomic hybridization (CGH). The first case was an unbalanced translocation t(7q;21q)(q34;q22.13) reported previously [24], whereas the second one was a deletion of 7q36. Both cases were featured by characteristic facial dysmorphisms, intellectual disability, and lumbosacral dysgenesis. Using array CGH and fluorescence *in situ* hybridization (FISH), we have narrowed the region of chromosome 7 (7q36.2q36.3 spanning from ~152 Mb to ~158 Mb) associated with common phenotypic features in these two cases. Using the present bioinformatic approach, *LIMBR1* and *MNX1* have been prioritized as candidates for lubosacral dysgenesis out of 45 genes located within the chromosome 7q36.2q36.3 region. Previously, we have confirmed candidate genes involved in chromosome abnormalities within a set of individuals suffering from intellectual disability, autism, epilepsy and/or congenital anomalies. The gene list has been provided in our previous studies [15,16]. In addition to previous data, candidate processes for these conditions were proposed: DNA replication, DNA damage and repair, nucleotide excision/mismatch repair, DNA damage-ATM-p53-apoptosis pathway, p53-/MAPK-/ErbB-/PI3KAkt-signaling, G1 to S cell cycle control, MAPK signaling pathway, mitotic cell cycle G1/S transition DNA damage checkpoint, p53-Dependent G1 DNA damage response and V(D)J recombination, axon guidance. Interestingly, an analysis of genome-wide associations studies in the light of somatic genomics of brain diseases has shown genes implicated in these pathways to be involved in the pathogenesis, as well [12]. Taken together, the approach seems to provide a possibility for prioritizing not only candidate genes according to (molecular) cytogenetic data but also alterations to molecular/cellular pathways and, thereby, candidates processes.

Naturally, the first filter in a molecular cytogenetic analysis is the chromosome abnormality or CNV itself, inasmuch as it narrows the search of candidates to a chromosomal region or even to a single gene. Computational genome annotation in cytogenetic analysis provides a possibility of associations between genes and manifestations of chromosomal imbalances [25]. However, such associations require additional molecular cytogenetic studies in larger cohorts or further molecular analyses of suggested candidate genes. The apparent lack of success in mapping genes of complex diseases (regardless of data on myriads of genetic variants associated with) evidences that, rather than gene hunting, specific changes in molecular/cellular processes should be considered as targets for the research and therapeutic interventions. In acknowledging this issue, an opportunity for uncovering disease pathways on the basis of molecular cytogenetic data is likely to be an important technological milestone.

Another implication of *in silico* molecular cytogenetics is the identification of regional genomic architecture leading to susceptibility to the formation of genome/chromosome rearrangements [26,27]. The capability of the present approach to acquire such data [15,24] would be useful for molecular cytogenetic research and diagnosis allowing the prediction of germline and somatic chromosomal/genomic rearrangements [28]. Thus, *in silico* molecular cytogenetics of chromosome imbalances should include the analysis of genomic databases for identification of regional genomic architecture in addition to gene prioritization.

### CNV prioritization

**Figure 3:**
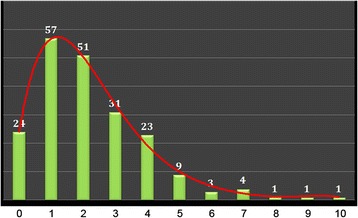
**CNV prioritization (abscissa: amount of CNV prioritized in each individual; ordinates: numbers of patients with the corresponding amount of CNV prioritized).** CNV detection was made by array CGH.

The determination of CNV pathogenicity can be designated as CNV prioritization. A phenotypic outcome has been proposed as the main criterion for CNV prioritization. To identify potential phenotypic effects of CNV by the present approach, the presence of at least one prioritized gene has been considered a criterion for the prioritization. The distribution of prioritized CNV detected by array CGH in the cohort (n = 205) is shown in Figure 3. In total, 462 CNV were prioritized in 181 patients giving a potential diagnostic yield of as high as 88.3%. It is to note that the distribution remotely resembles the normal (or Gaussian) distribution. This has allowed us to speculate that amount of causative CNV per patient has a tendency to vary generally between 1 and 3 in clinical cohorts of patients with neurodevelopmental (brain) diseases (Figure 3).

The problem of differentiating between pathogenic and benign CNV seems to remain actual [7,8,16,25]. Furthermore, better understanding of genetic variation and its relevance to mechanisms of neurodevelopmental diseases has been originated from application of high-throughput genome analyses [29]. Although there has been described several ways to process the data for identification of disease mechanisms [8,29], interpretation of a patient’s variome represents a challenge. Indeed the success of such analysis can give valuable information for understanding disease mechanisms and, as a result, suggest therapeutic interventions. Furthermore, CNV prioritization in larger cohorts might indicate as candidate disease processes as molecular and cellular pathways implicated in complex biological phenomena (i.e. aging, cell cycle control, transcriptional regulation, chromatin remodeling, genome stability maintenance) [12,30-32].

### *In silico* molecular cytogenetic analysis of somatic mosaicism

To test the possibility of developing an *in silico* molecular cytogenetic method for evaluating somatic genome variations (SGV), we have processed the data on interphase and metaphase FISH analysis of about 5000000 cells from 150 samples. SGV were analyzed in two ways: (i) evaluation of SGV consequences in terms of cellular or clinical phenotype [14]; (ii) analysis of variome (non-mosaic variations in an individual genome) to hypothesize SGV origins [12]. The former way was mainly used for structural genomic variations and genomic instability (GIN) while the latter way was used for analyzing origins of chromosome instability (CIN) or aneuploidy/polyploidy [12,33]. In the set of samples analyzed by FISH-based techniques, aneuploidy has been the most common type of mosaic genome variations. Since aneuploidy represents one of the most devastating types of SGV, affecting from hundreds to thousands genes and impacting on cellular phenotype [34,35], consequences of aneuploidization were not suggested to require further bioinformatic evaluations in the phenotypic context. Moreover, the effect of chromosome number variations has been recently modeled for assessing somatic genome evolution in cancer showing elevated tolerance to aneuploidy or, in other words, global changes to genomic (epigenomic) landscape [36]. However, GIN and CIN manifested as chromosome breaks and rearrangements were found to be appropriate for finding genomic loci susceptible to breakage (i.e. chromosomal fragile sites and chromosomal regions containing highly repetitive DNA) and mapping genes disrupted by CIN in brain disease (ataxia-telangiectasia and Alzheimer’s disease) (for more details see [14,37]). It is noteworthy that these genomic changes cannot be detected by high-throughput technologies of whole genome analysis. Therefore, such types of GIN/CIN are rarely evaluated by an *in silico* analysis, even though knowledge of their effects on cellular/clinical phenotypes is able to shed light on new genetic mechanisms of biodiversity and disease [38]. Alternatively, it has been shown that either SGV or non-mosaic genomic variations can dysregulate chromosome segregation and genomic maintenance producing CIN or GIN [12]. These observations were used for suggesting that bioinformatic approaches might be useful for studying mechanisms and consequences of somatic mosaicism. Finally, the present approach based on prioritizing genetic variants using evaluation of epigenetic variation between tissues and cell types can be utilized in studies of tissue-specific mosaicism.

Until recently, somatic mosaicism has not been a major focus of genome research [33,39]. With the increase of interest in addressing SGV, several studies have posed questions about the relevance of SGV to genetic diversity and morbidity [39-41]. It is repeatedly noted that SGV are underrecognized sources of genomic, chromosomal and complex disorders [18,33,39,41]. Additionally, SGV affecting specific tissues often lead not only to cancer, but also to tissue-specific pathology. For instance, numerous brain diseases are associated with SGV (CIN/GIN) manifested as aneuploidy or structural genome variations [14,18,37,42-48]. These data have served as a basis for speculations about diagnostic applications of SGV analysis in brain disease and regeneration therapy [49]. Still, the idea remains undeveloped and further theoretical input is needed. It can be expected that molecular cytogenetic studies of SGV would benefit from *in silico* evaluations of their mechanisms and consequences.

## Conclusions

Bioinformatics can help in avoiding extensive laboratory efforts, but requires deep knowledge in computer science, statistics and related disciplines. Nevertheless, the existence of user-friendly online tools and software is able to simplify the use of bioinformatics. Here, a bioinformatic approach to prioritization of candidate genes and CNV based on analysis of genomic/epigenetic/proteomic and metabolomic databases/online tools has been proposed. According to our evaluations, it seems that the approach possesses simplicity inasmuch as it does not require sophisticated computational or statistical methodology. Another advantage of the present bioinformatic approach is the versatility or, more precisely, the independence from the use of specific software and databases (online tools).

**Figure 4:**
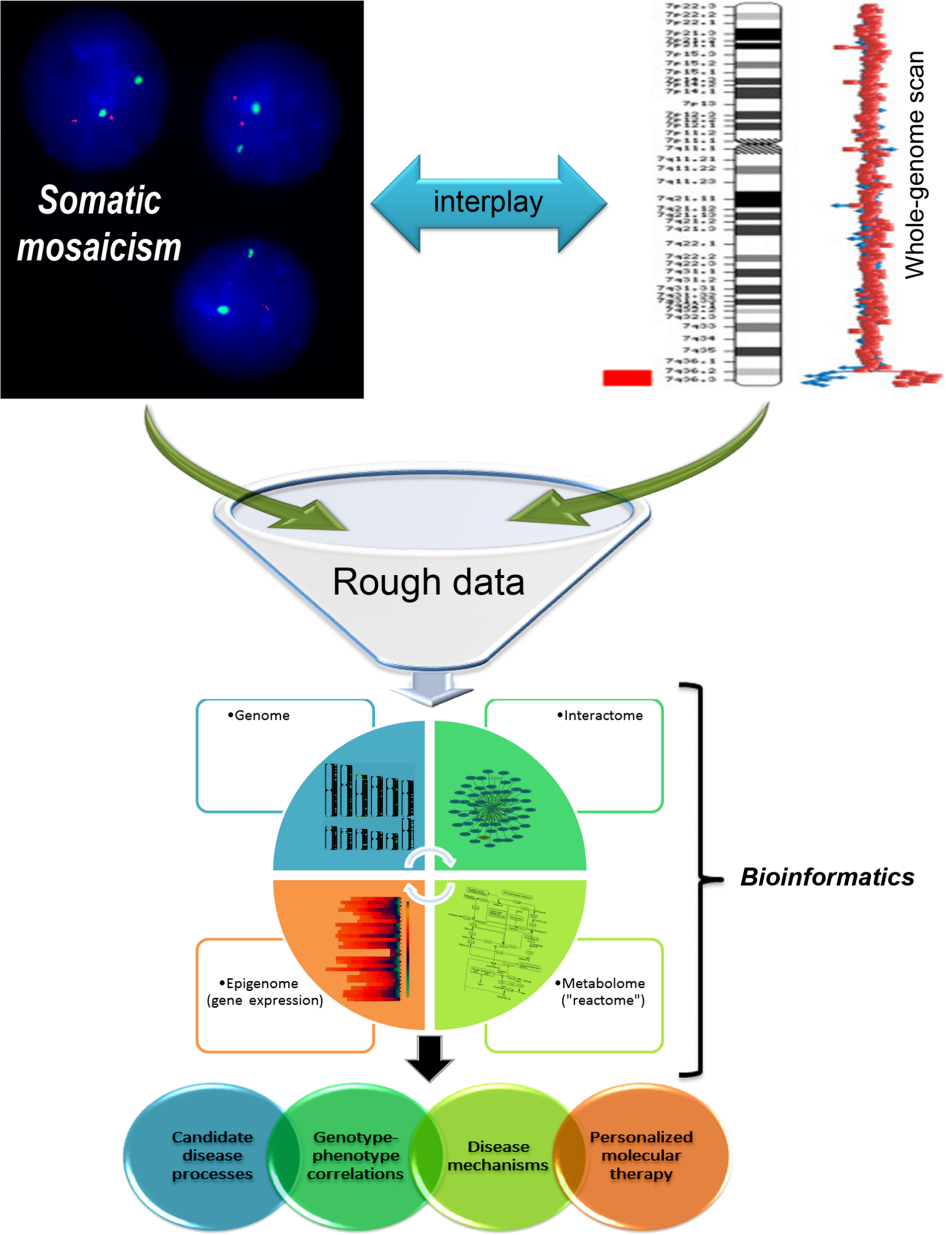
***In silico*** **molecular cytogenetics (flow chart of the approach).** Molecular cytogenetic data (genome data) acquired through techniques for whole-genome scan (i.e. array CGH) and detecting SGV (i.e. interphase FISH) is analyzed by the bioinformatic approach (genome, epigenome, interactome and metabolome or “reactome” analysis), which is able not only to define interplay between mosaicism, CIN and GIN with heritable/*de novo* (non-mosaic) genomic variations, but also to identify candidate disease processes allowing appropriate genotype-phenotype correlations and, thereby, determination of intrinsic disease mechanisms. The latter has the potential to become a basis for successful personalized molecular therapy (scheme was partially inspired by [10,28,52]).

Combining molecular cytogenetic resolution in whole-genome scanning and single-cell chromosomal analysis [50-52] with the power of bioinformatic analyses of transcriptomic and proteomic data [53-55], the approach is able to shed light on interplay between SGV and non-mosaic chromosomal/genomic rearrangements. Further, filtering and ranking genomic, epigenomic (gene expression), interactomic (protein networks) and metabolomics (“reactome”/pathways) data followed by the fusion forms the basis for prioritizing candidate disease processes. These are certainly useful for making genotype-phenotype correlations, elucidating disease mechanisms, and developing personalized molecular therapy. Taken together, these theoretical perspectives provide the foundation of *in silico* molecular cytogenetics, basic principles (outline) of which are schematically illustrated in Figure 4. It is to note that this scheme is a kind of flow chart of our bioinformatic approach, as well. To this end, one can still conclude that further steps to make *in silico* molecular cytogenetics more practical are certainly required.

## Methods

### Molecular cytogenetic techniques (sample preparation, FISH and array CGH)

We have analyzed the results of array CGH of 205 patients. Additionally, data on interphase FISH analysis of about 5000000 cells from 150 samples (metaphase and interphase analyses) were bioinformatically evaluated. Array CGH was performed as described previously [16,56]. FISH analyses of metaphase chromosomes were presented earlier [44,57]. Sample preparation for interphase FISH was made according to a previously described protocol [58]. Results of interphase and metaphase FISH with chromosome numeration, site-specific and multicolor banding DNA probes were described in our previous communications [14,37,42,44,45,48,52,57].

### Bioinformatics

Genomic, epigenomic, proteomic and metabolomic data was analyzed as described previously [1-6,12,14-16,59]. The data on each gene involved in chromosome abnormalities and/or CNV were acquired from clinical, genomic (browsers and gene ontology databases), epigenetic (gene expression), proteomic, interactomic (databases + software) and metabolomic databases. Firstly, genes were selected according to the gene expression patterns. Proteomic and metabolomic data were used to confirm the selection. More precisely, epigenetic (expression) and metabolic “tissue-specificity” was used as a criterion for the selection (for more details see [17] and [59]). Interactomic data was visualized and processed using Cytoscape software [60]. Metabolomic data was acquired from multiple sources (i.e. gene ontology databases and the Reactome pathway knowledgebase [61]).

The prioritization was made by ontology-based gene filtering (i.e. selection of genes according to their direct relevance to the phenotype or to their involvement in molecular/cellular processes relevant to a trait). Afterwards, ontology-based gene-specific ranking of gene properties was used. To finalize the prioritization, simulating pathway alterations (i.e. analyzing proteomic networks without elements referred to mutated/deleted/duplicates genes) was made and then, the selection of candidate processes for pathology in each given case was done. The prioritization of one or more genes in a CNV encompassing several genes was considered as an essential criterion for the prioritization. Information from clinical and molecular databases was arbitrarily re-evaluated by a bibliographic analysis assessing the level of publications (citations, comments etc.) about features of interest in light of the phenotypic outcome.

### Databases and software

Databases and software used in the present study are outlined in Table 1.

**Table 1:** **Databases, tools, resources and software used in the present study**

**Database-tool-resource-software**	**URL**	**Acquired data or application**
UCSC Genome Browser (Version: Feb. 2009 GRCh37/hg19)	http://genome.ucsc.edu/	Mapping of molecular cytogenetic data
Ensembl Genome Browser	http://www.ensembl.org/index.html	
NCBI Build 37.1/NCBI Map Viewer	http://www.ncbi.nlm.nih.gov/projects/mapview/map_search.cgi?taxid=9606	
Database of Genomic Variants	http://dgvbeta.tcag.ca/dgv/app/home?ref=GRCh37/hg19	Data on natural genome variations
OMIM (online Mendelian inheritance in Man)	http://www.omim.org/	Clinical data
DECIPHER (Database of Chromosomal Imbalance and Phenotype in Humans using Ensembl Resources)	http://decipher.sanger.ac.uk/	
Phenotype-Genotype Integrator (PheGenI)	http://www.ncbi.nlm.nih.gov/gap/PheGenI	
AutDB (web-based searchable database for autism research)	http://www.mindspec.org/autdb.html	
BioGPS	http://biogps.org [17]	Gene expression data
Cytoscape software (Version: 3.1.1)	http://www.cytoscape.org/ [60]	Interactome analysis
Reactome	http://www.reactome.org/ [61]	Pathway analysis
Pathway commons	http://www.pathwaycommons.org	
KEGG (Kyoto Encyclopedia of Genes and Genomes)	http://www.genome.jp/kegg/	
NCBI BioSystems Database	http://www.ncbi.nlm.nih.gov/biosystems	
NCBI gene	http://www.ncbi.nlm.nih.gov/gene/	Various gene information
PubMed	http://www.ncbi.nlm.nih.gov/pubmed/	Bibliographic searches and evaluations
Google scholar	http://www.scholar.google.com/	

## Abbreviations

CGH: Comparative genomic hybridization
CIN: Chromosome instability
CNV: Copy number variations
FISH: Fluorescence *in situ* hybridization
GIN: Genome instability
SGV: Somatic genome variations
URL: Uniform Resource Locator
